# Supplementary material for: Detecting recurrent passenger mutations in melanoma by targeted UV damage sequencing
Source: Nat Commun. 2023 May 11;14:2702. doi: 10.1038/s41467-023-38265-3 (PMC10175485; doi:10.1038/s41467-023-38265-3)
Supplement: Supplementary file 1 — Supplementary Information [file 41467_2023_38265_MOESM1_ESM.pdf]

Supplementary Information

Title: Detecting recurrent passenger mutations in melanoma by targeted UV damage sequencing

Selvam et al.

Supplementary Table 1 | Location, function, and CPD lesion count of candidate non-coding driver mutations in melanoma.

Mutation location	Mutation count[a]	CPD count[b]	Gene	Function
chr5:1295228	14	0	TERT	Telomerase reverse transcriptase; frequently mutated in many cancers
chr5:1295242	15	0		
chr5:1295250	20	2		
chr3:67048644	8	0	KBTBD8	Neural crest and melanocyte differentiation
chr20:36156403	10	12	BLCAP	Apoptosis inducing factor linked to bladder cancer
chr1:153963227	13	78[c]	RPS27	Component of the small ribosomal subunit
chr3:52029960	11	113	RPL29	Component of the large ribosomal subunit
chr22:31556121	10	60	RNF185	E3 ubiquitin-protein ligase, endoplasmic reticulum-associated degradation (ERAD) pathway
chr16:89284086	7	245	ZNF778	Zinc Finger Protein, transcriptional regulation?
chr20:32580927	23	184	RALY	Pre-mRNA splicing
chr20:32581032	5	642		
chr10:18940601	5	1112	NSUN6	tRNA C5-cytosine methylation

[a]Mutation data from Hayward et al., 2017[1].
[b]CPD count derived from CPD-capture-seq reads from UVB-irradiated melanocytes.
[c]All mutations/rows with shading are predicted to be recurrent passenger mutations due to CPD hotspot associated with the mutation site, based on CPD-capture-seq data

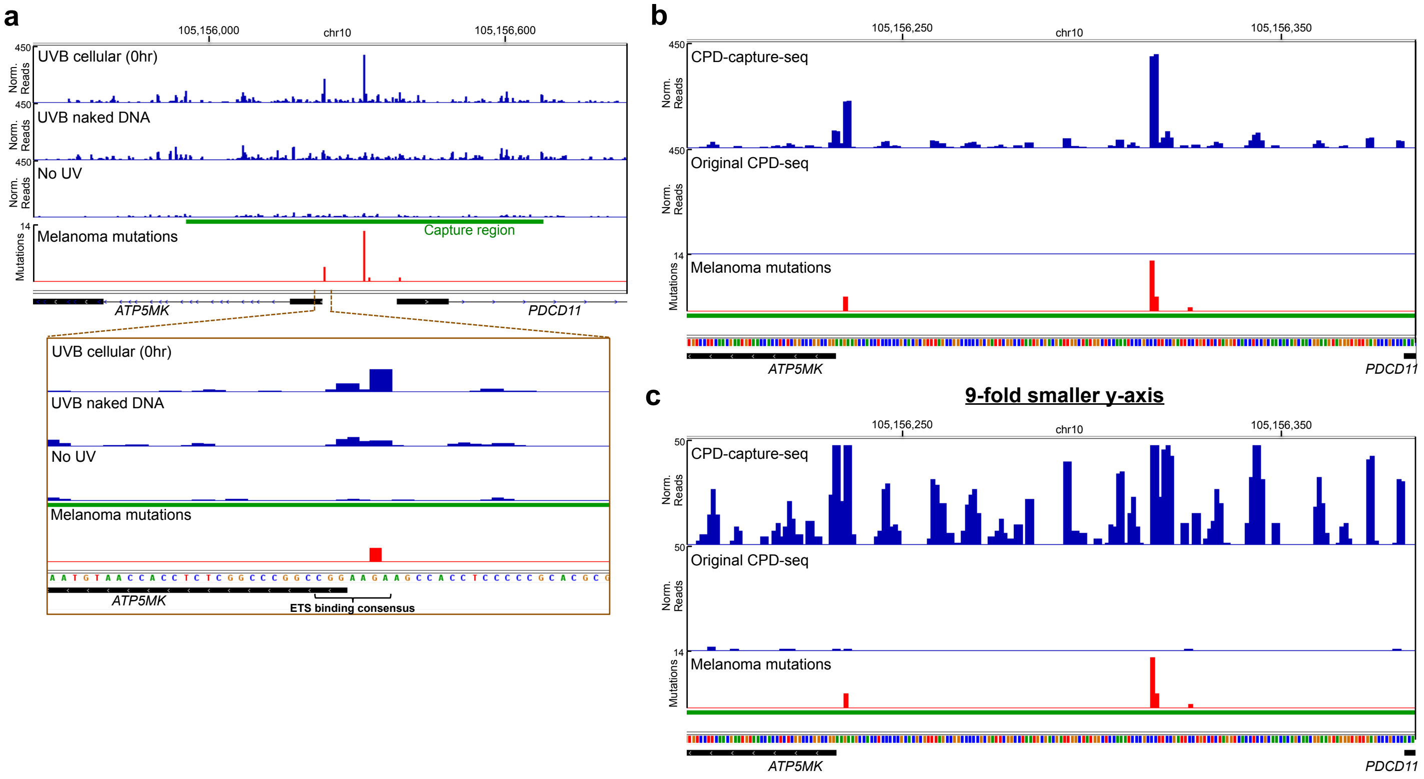

Supplementary Fig. 1 | Close up view of CPD-capture-seq and original CPD-seq data for the *PDCD11* and *ATP5MK* promoter. a, Same as Fig. 1f, except zoomed on second mutation hotspot near the *ATP5MK* gene. **b-c**, Comparison of CPD-capture-seq read density with the density of reads from four original CPD-seq libraries derived from UVC-irradiated NHF1 cells[1]. Panel **c** shows a zoomed scale, so that many of the peaks in the CPD-capture-seq lane are off the scale. Figure generated using IGV[2].

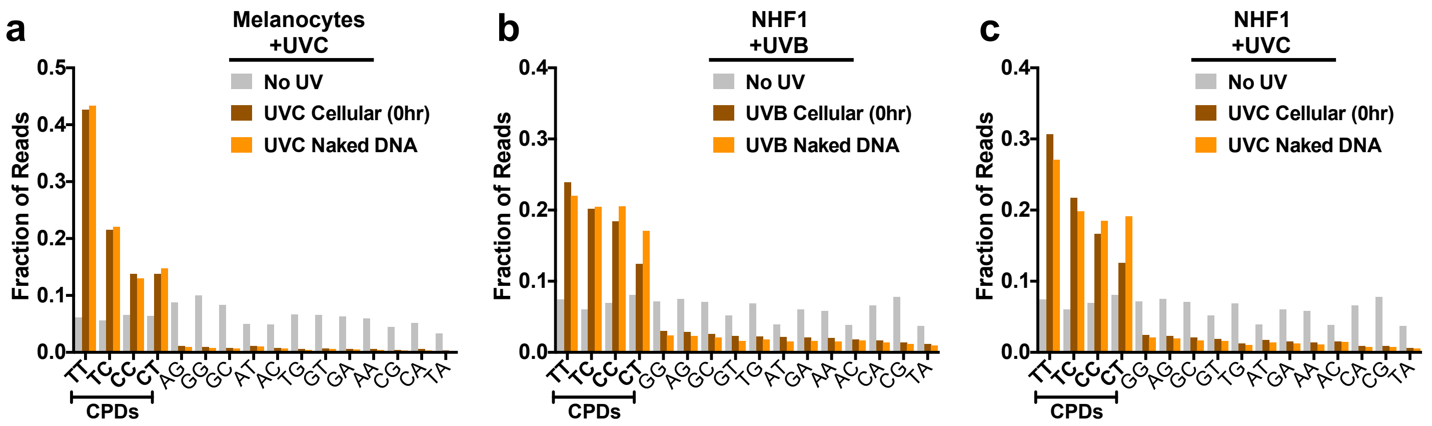

Supplementary Fig. 2 | CPD-capture-seq reads in UV-irradiated samples are enriched at CPD-forming dipyrimidine sequences. a-c, Fraction of CPD-capture-seq reads associated with a putative lesion at the indicated dinucleotide in UVB- or UVC-irradiated NHF1 cells or melanocytes. Source data for graphs in panels **a**-**c** are provided as a Source Data file.

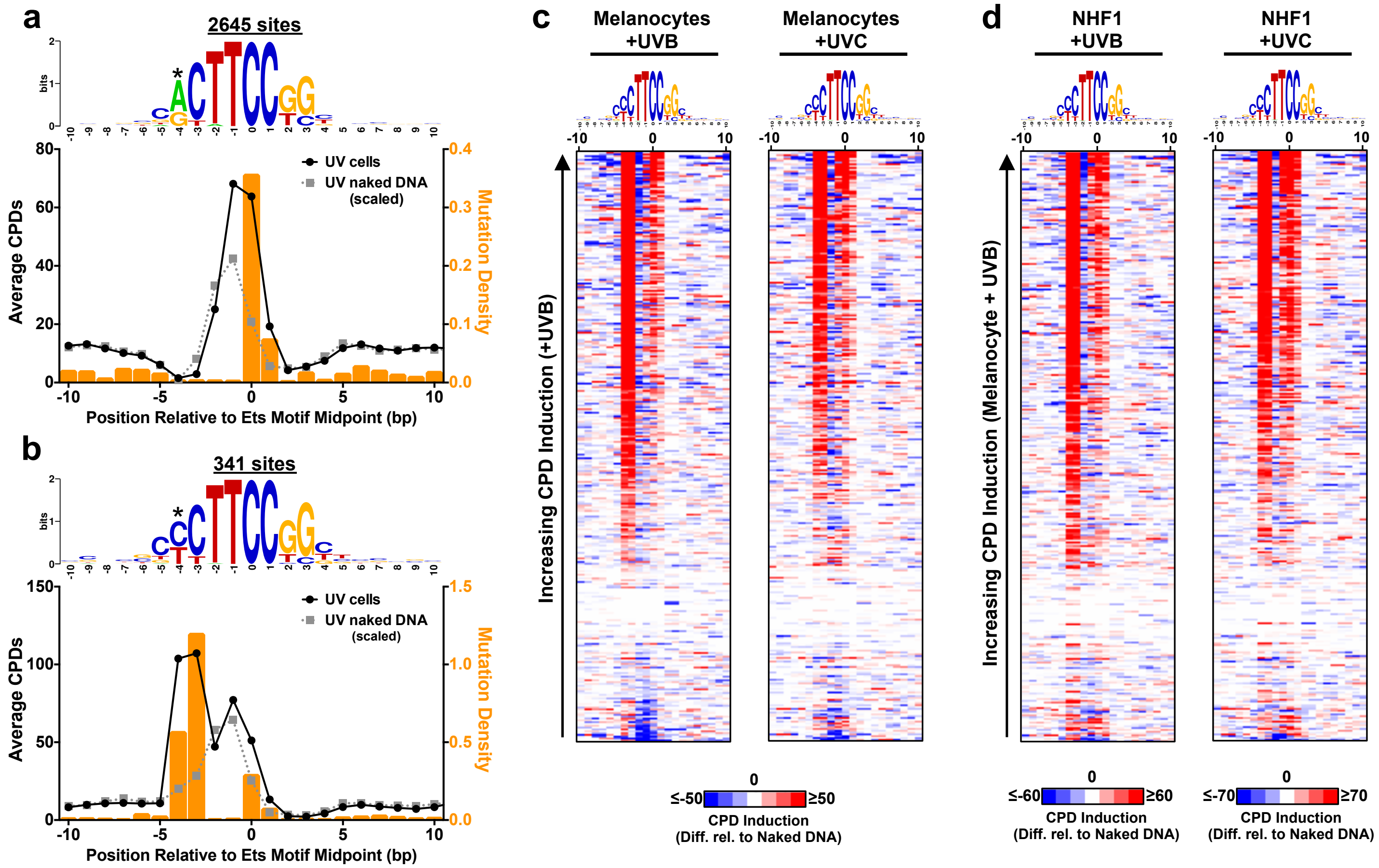

Supplementary Fig. 3 | UV damage induction at ETS binding sites in the UVC-irradiated melanocytes. a-b, Graphs of average CPD density and somatic mutations in (**a**) canonical (no dipyrimidine at positions -3/-4) or (**b**) variant (dipyrimidine at positions -3/-4) ETS binding sites. Only active ETS binding sites (defined as binding sites for ELK4, ETS1, and GABPA from ENCODE that are present in a melanocyte DNase I hypersensitivity site were analyzed. Somatic mutation data was from 183 sequenced melanoma genomes (ICGC). Sequence logo was created using weblogo[3]. **c-d**, Cluster analysis of CPD-capture-seq data for each individual variant ETS binding site (i.e., dipyrimidine at positions -3/-4 relative to the motif midpoint). CPD induction, defined as the difference in CPD levels between the UV-irradiated cells and the scaled naked DNA control, is depicted (see color bar). Binding sites were ordered by increasing CPD induction in the ETS motif in the UVB-irradiated melanocyte sample. Source data for graphs in panels **a** and **b** are provided as a Source Data file.

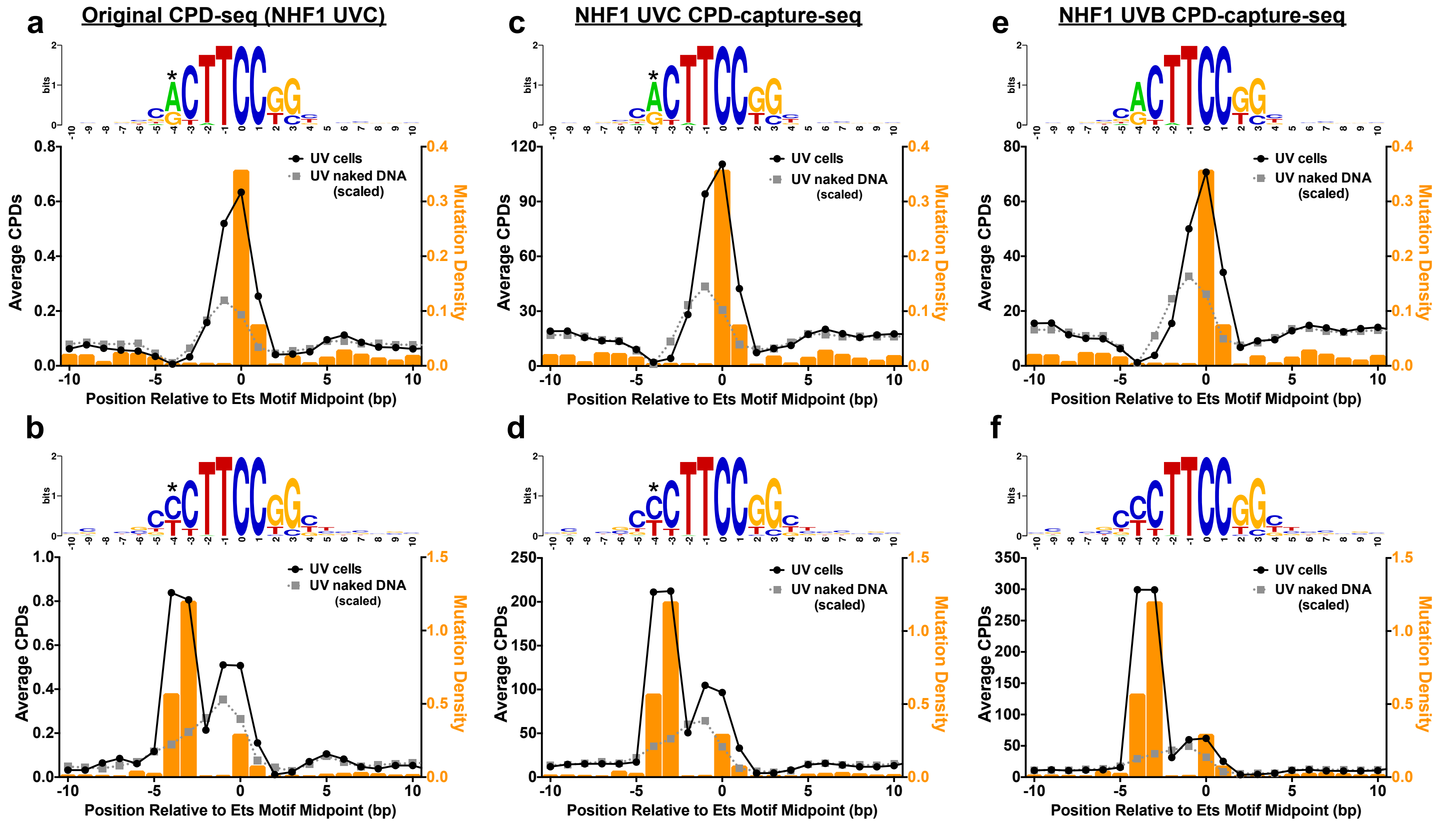

Supplementary Fig. 4 | CPD damage induction at ETS binding sites in immortalized normal human skin fibroblasts (NHF1 cells). a-f, Same as Supplementary Fig. 3a,b, except for NHF1 cells irradiated with UVC or UVB. (**a-b**) Analysis of original CPD-seq data[1,4]. (**c-d**) Analysis of CPD-capture-seq data for UVC-irradiated NHF1 cells. **(e-f**) Analysis of CPD-capture-seq data for UVB-irradiated NHF1 cells. Source data for graphs in panels **a-f** are provided as a Source Data file.

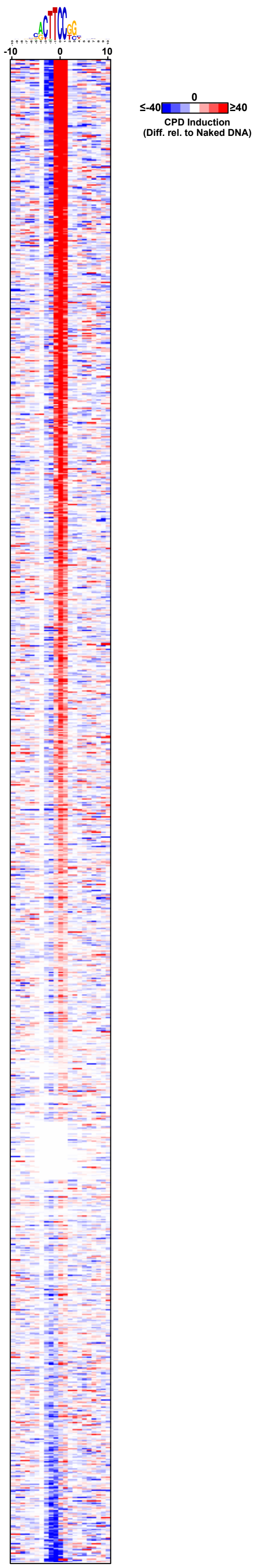

Supplementary Fig. 5 | UV damage induction is associated with a subset of canonical ETS binding sites in primary melanocytes. Cluster analysis of CPD-capture-seq data for each individual canonical ETS binding site (i.e., no dipyrimidine at positions -3/-4 relative to the motif midpoint). Binding sites were ordered by increasing CPD induction in the ETS motif, defined as the difference in CPDs in the UVB-irradiated melanocyte sample relative to the scaled UVB-irradiated naked DNA control.

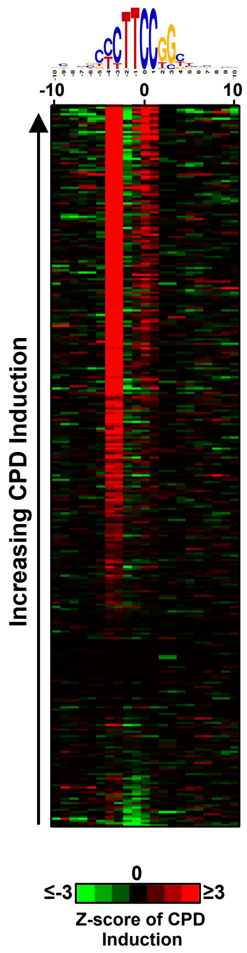

Supplementary Fig. 6 | CPD-capture-seq reads are significantly elevated at ETS binding sites. Same as Fig. 2c (left panel), except Z-score of CPD induction is depicted.

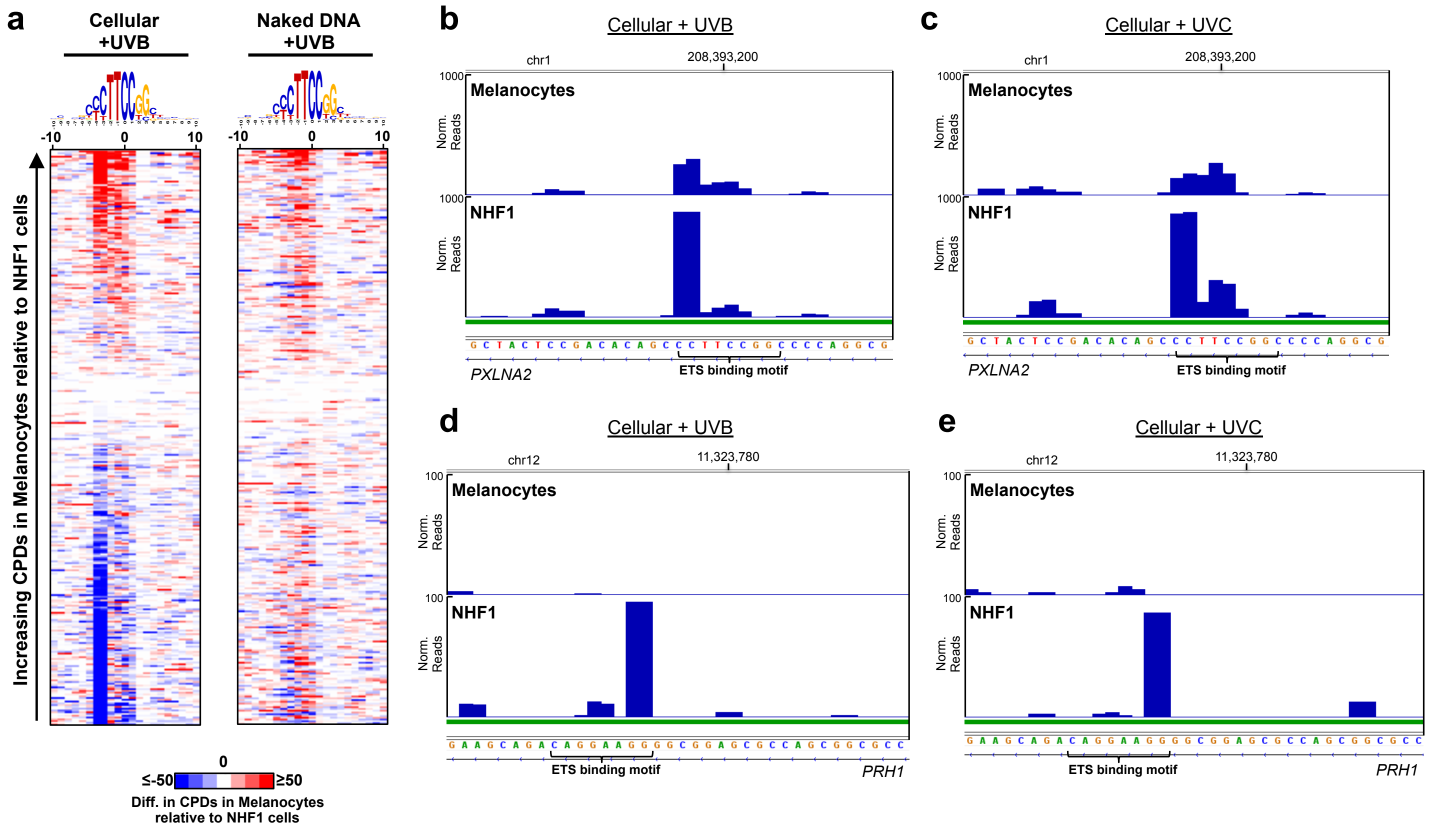

Supplementary Fig. 7 | UV damage induction at subset of ETS binding sites differs in primary melanocytes and skin fibroblasts. a, Cluster plot showing difference in CPD levels at variant ETS binding sites (-3/-4 positions are a dipyrimidine) in UVB-irradiated primary melanocytes relative to scaled UVB-irradiated NHF1 cells (left panel). Right panel shows difference in CPD levels between UVB-irradiated melanocyte genomic DNA and NHF1 genomic DNA as a control. Both panels have the same order, with increasing CPDs in melanocytes relative to NHF1 cells. Sequence logos generated using weblogo[3]. **b-e**, Closeup of differential UV damage levels at ETS binding sites associated with introns in the genes (**b,c**) *PXLNA2* and (**d,e**) *PRH1*. Normalized CPD-capture-seq reads are shown for primary melanocytes and NHF1 cells following **(b,d**) UVB- or (**c,e**) UVC-irradiation. For both of these binding sites, consistently higher CPD levels are associated with UV-irradiated NHF1 cells, likely reflecting higher ETS TF binding in NHF1 cells. Images generated using IGV[2].

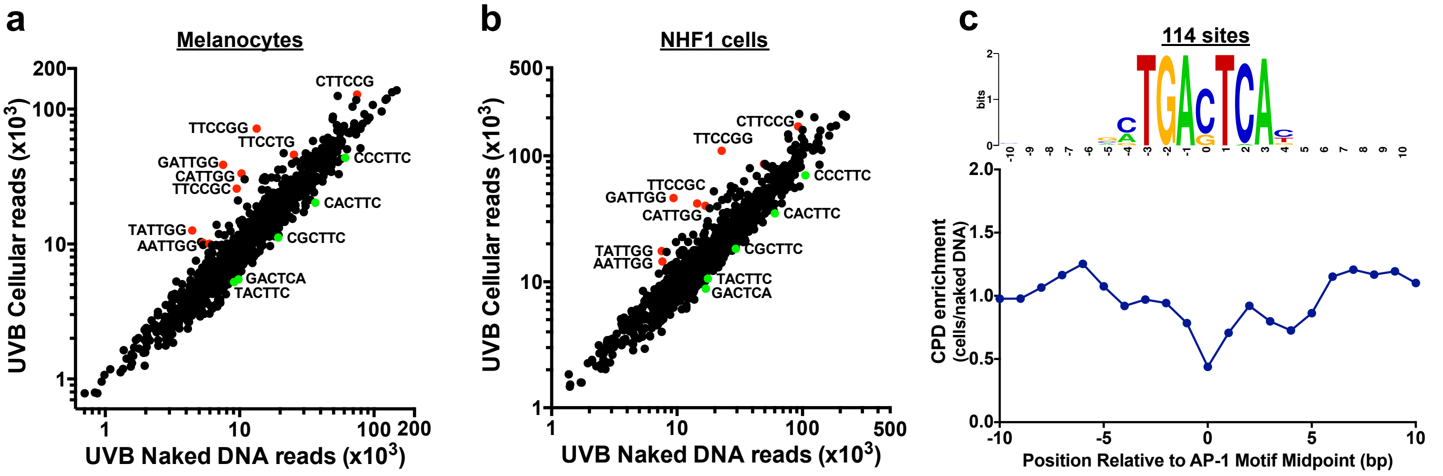

Supplementary Fig. 8 | UV-induced CPD formation is modulated at specific hexamer sequences, many of which are associated with TF binding sites. a-b, Count of CPD-capture-seq reads in UVB-irradiated cellular or naked DNA samples in different hexamer sequence contexts. Data for UVB-irradiated (**a**) melanocytes and (**b**) NHF1 cells are shown. **c,** Normalized enrichment of CPDs at active Fos/Jun (AP-1) sites that overlap with a capture region in UVB-irradiated melanocytes relative to UVB-irradiated naked DNA. Sequence logo of binding sites is shown in top panel, and was made using weblogo[3]. Source data for graphs in panels **a-c** are provided as a Source Data file.

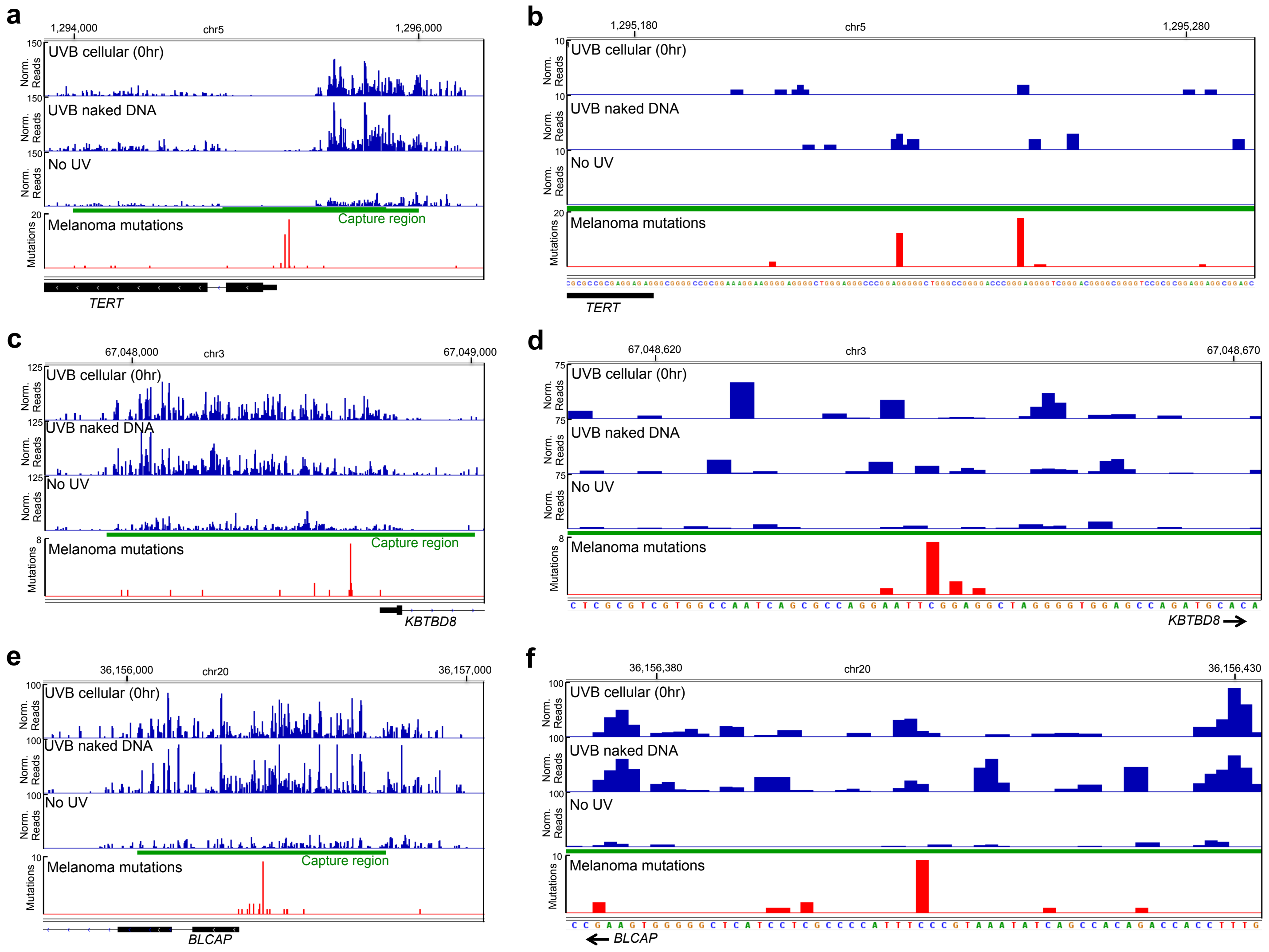

Supplementary Fig. 9 | Candidate non-coding driver mutations in the promoters of *TERT*, *KBTBD8*, and *BLCAP* are not associated with elevated UV damage levels in UVB-irradiated primary melanocytes. a-b, Views of normalized CPD-capture-seq data (at lesion-forming dipyrimidines) for UVB-irradiated melanocytes, UVB-irradiated naked DNA, and no UV control at the *TERT* promoter. Mutation data from 183 melanomas (ICGC) is shown for reference. **c-d**, Same as panels **a-b**, except for *KBTBD8* promoter. **e-f,** Same as panels **a-b**, except for *BLCAP* promoter. Images generated using IGV[2].

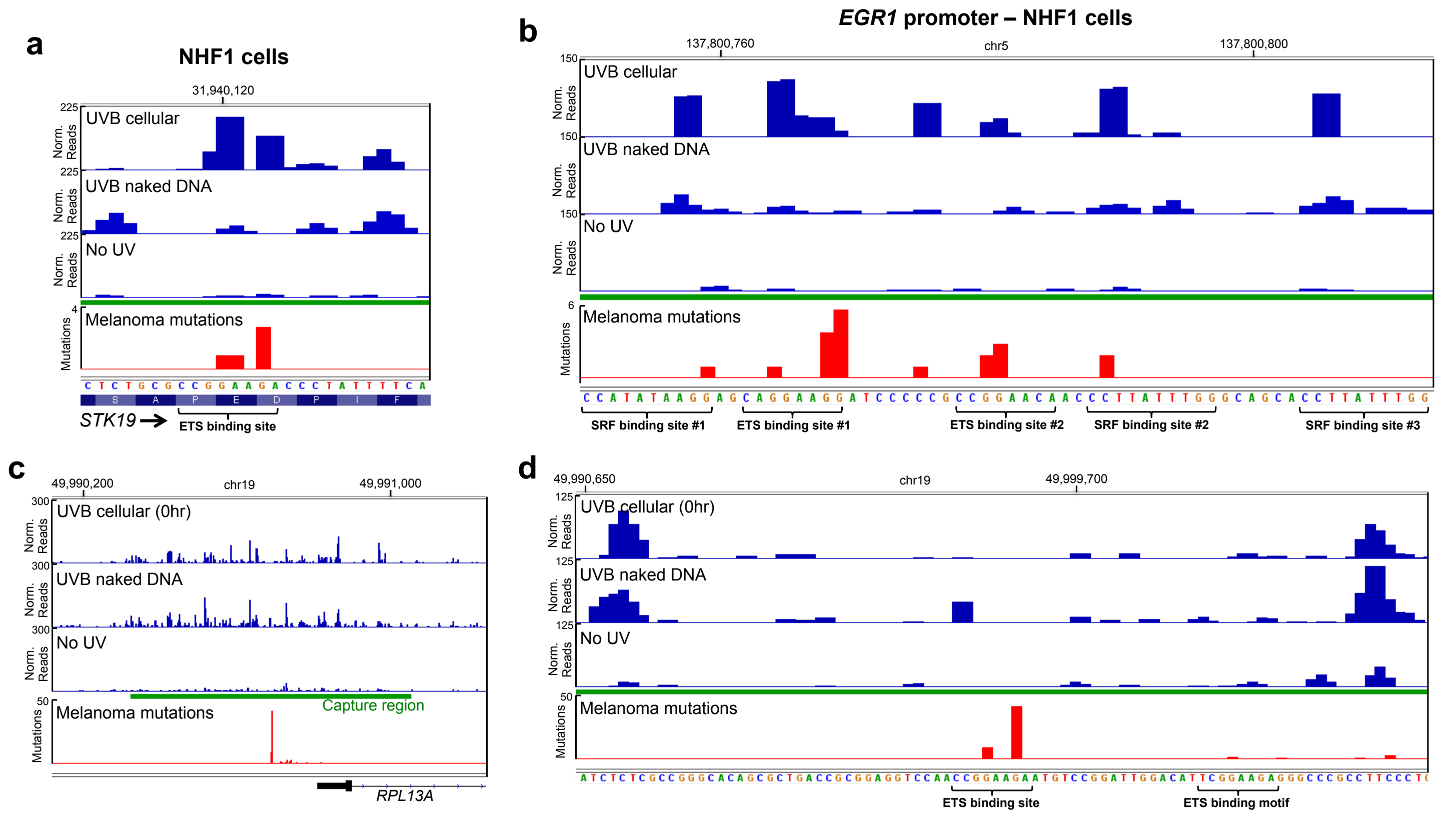

Supplementary Fig. 10 | UV damage formation in *STK19* gene and *EGR1* promoter in NHF1 cells and *RPL13A* promoter in melanocytes. a, UV damage is induced at *STK19* D89 codon in UVB-irradiated NHF1 cells. Views of normalized CPD-capture-seq data (at lesion-forming dipyrimidines) for UVB-irradiated NHF1 cells, UVB-irradiated naked DNA, and no UV control. Mutation data from 183 melanomas (ICGC) is shown for reference. **b**, Same as panels **a**, except for *EGR1* promoter. **c-d,** Same as panels **a**, except for UVB-irradiated primary melanocytes at the *RPL13A* promoter. Images generated using IGV[2].

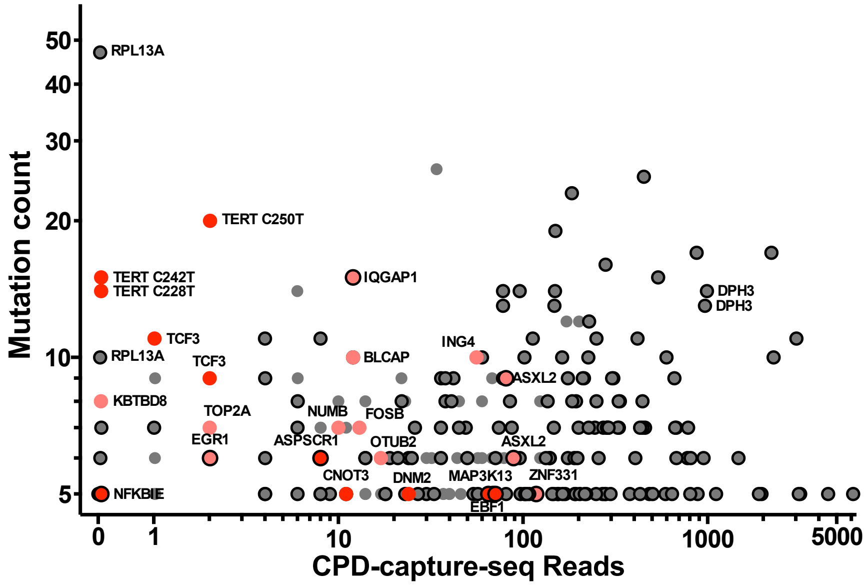

Supplementary Fig. 11 | CPD density in UV-irradiated melanocytes at sites of recurrent promoter mutations. Same as Fig. 4a, except showing sites with at least 5 mutations. Promoter mutations associated with known or suspected cancer genes are indicated in red and salmon color, respectively. Black outline indicates that the mutation occurs in an ETS binding site. Source data are provided as a Source Data file.

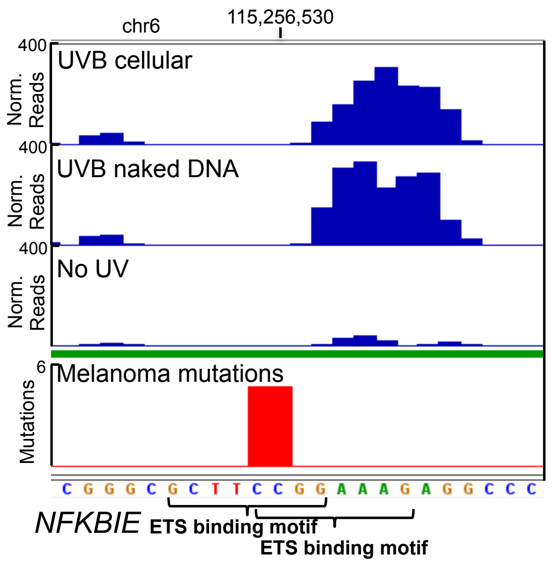

Supplementary Fig. 12 | UV damage is not elevated at recurrent mutation in *NFKBIE* gene. Views of normalized CPD-capture-seq data (at lesion-forming dipyrimidines) for UVB-irradiated melanocytes, UVB-irradiated naked DNA, and no UV control at the *NFKBIE* gene. Protein-coding translation for *NFKBIE* is not shown because the default gene model in IGV does not include this genomic region. Images generated using IGV[2].

References

1. Mao, P. *et al.* ETS transcription factors induce a unique UV damage signature that drives recurrent mutagenesis in melanoma. *Nat Commun* **9**, 2626 (2018).

2. Thorvaldsdottir, H., Robinson, J. T. & Mesirov, J. P. Integrative Genomics Viewer (IGV): high-performance genomics data visualization and exploration. *Brief Bioinform* **14**, 178-192 (2013).

3. Crooks, G. E., Hon, G., Chandonia, J. M. & Brenner, S. E. WebLogo: a sequence logo generator. *Genome Res* **14**, 1188-1190 (2004).

4. Sivapragasam, S. *et al.* CTCF binding modulates UV damage formation to promote mutation hot spots in melanoma. *Embo j* **40**, e107795 (2021).
