# Supplementary material for: Detecting recurrent passenger mutations in melanoma by targeted UV damage sequencing
Source: Nat Commun. 2023 May 11;14:2702. doi: 10.1038/s41467-023-38265-3 (PMC10175485; doi:10.1038/s41467-023-38265-3)
Supplement: Supplementary file 3 — Reporting Summary [file 41467_2023_38265_MOESM3_ESM.pdf]

nature portfolio

Corresponding author(s): John Wyrick

Last updated by author(s): Apr 14, 2023

Reporting Summary

Nature Portfolio wishes to improve the reproducibility of the work that we publish. This form provides structure for consistency and transparency in reporting. For further information on Nature Portfolio policies, see our Editorial Policies and the Editorial Policy Checklist.

Statistics

For all statistical analyses, confirm that the following items are present in the figure legend, table legend, main text, or Methods section.

n/a	Confirmed	
☐	☒	The exact sample size (*n*) for each experimental group/condition, given as a discrete number and unit of measurement
☐	☒	A statement on whether measurements were taken from distinct samples or whether the same sample was measured repeatedly
☐	☒	The statistical test(s) used AND whether they are one- or two-sided<br>*Only common tests should be described solely by name; describe more complex techniques in the Methods section.*
☒	☐	A description of all covariates tested
☒	☐	A description of any assumptions or corrections, such as tests of normality and adjustment for multiple comparisons
☐	☒	A full description of the statistical parameters including central tendency (e.g. means) or other basic estimates (e.g. regression coefficient) AND variation (e.g. standard deviation) or associated estimates of uncertainty (e.g. confidence intervals)
☐	☒	For null hypothesis testing, the test statistic (e.g. *F*, *t*, *r*) with confidence intervals, effect sizes, degrees of freedom and *P* value noted<br>*Give P values as exact values whenever suitable.*
☒	☐	For Bayesian analysis, information on the choice of priors and Markov chain Monte Carlo settings
☒	☐	For hierarchical and complex designs, identification of the appropriate level for tests and full reporting of outcomes
☒	☐	Estimates of effect sizes (e.g. Cohen's *d*, Pearson's *r*), indicating how they were calculated

Our web collection on statistics for biologists contains articles on many of the points above.

Software and code

Policy information about availability of computer code

Data collection	None
Data analysis	Bowtie2, SAMtools, BEDTools, Excel (version 14.7.7) and custom Perl scripts were used to analyze CPD-capture-seq data. Data was analyzed (e.g., Poisson regression) or plotted using Graphpad Prism (version 6 and 8) or visualized using IGV. Custom Perl scripts, etc. are available at: https://github.com/bmorledge-hampton19/CPD-Capture-seq

For manuscripts utilizing custom algorithms or software that are central to the research but not yet described in published literature, software must be made available to editors and reviewers. We strongly encourage code deposition in a community repository (e.g. GitHub). See the Nature Portfolio guidelines for submitting code & software for further information.

Data

Policy information about availability of data

All manuscripts must include a data availability statement. This statement should provide the following information, where applicable:

- Accession codes, unique identifiers, or web links for publicly available datasets
- A description of any restrictions on data availability
- For clinical datasets or third party data, please ensure that the statement adheres to our policy

The CPD-capture-seq data described in this study is available under GEO accession: GSE225362. Human genome (hg19) is available at: https://hgdownload.soe.ucsc.edu/downloads.html DNase-seq data for skin melanocytes (E059) is available from GEO under the accessions GSM774243, GSM774244, and GSM1024610. Genome-wide somatic mutation data for 183 melanoma genomes is available from the International Cancer Genome Consortium (ICGC) (https://

dcc.icgc.org/releases/release_20/Projects/MELA-AU). Lists of recurrent mutations at promoters, protein-coding regions and 5' and 3'UTRs are available at https://www.nature.com/articles/nature22071.

Human research participants

Policy information about studies involving human research participants and Sex and Gender in Research.

Reporting on sex and gender	Not Applicable
Population characteristics	Not Applicable
Recruitment	Not Applicable
Ethics oversight	Not Applicable

Note that full information on the approval of the study protocol must also be provided in the manuscript.

Field-specific reporting

Please select the one below that is the best fit for your research. If you are not sure, read the appropriate sections before making your selection.

- [x] Life sciences
- [ ] Behavioural & social sciences
- [ ] Ecological, evolutionary & environmental sciences

For a reference copy of the document with all sections, see nature.com/documents/nr-reporting-summary-flat.pdf

Life sciences study design

All studies must disclose on these points even when the disclosure is negative.

Sample size	Sample sizes were chosen based on our prior experience with CPD seq (PMID: 29980679).
Data exclusions	No CPD-capture-seq data were excluded.
Replication	In vitro CPD damage mapping at STK19 region was replicated four times. All replication attempts were successful.<br>CPD-capture-seq experiments were not replicated. However, we have designed and compared independent experiments (4 total) with two different treatments (i.e., UVB or UVC) and two different cell types (i.e., melanocytes and NHF1 cells) to confirm our findings.
Randomization	Cell populations were exposed to different treatments (UVB or UVC, etc.) randomly.
Blinding	No - instead each data set was analyzed equally using the same bioinformatics pipeline irrespective of sample identity.

Reporting for specific materials, systems and methods

We require information from authors about some types of materials, experimental systems and methods used in many studies. Here, indicate whether each material, system or method listed is relevant to your study. If you are not sure if a list item applies to your research, read the appropriate section before selecting a response.

Materials & experimental systems

n/a	Involved in the study	
x		Antibodies
	x	Eukaryotic cell lines
x		Palaeontology and archaeology
x		Animals and other organisms
x		Clinical data
x		Dual use research of concern

Methods

n/a	Involved in the study	
x		ChIP-seq
x		Flow cytometry
x		MRI-based neuroimaging

Eukaryotic cell lines

Policy information about cell lines and Sex and Gender in Research

Cell line source(s)	Normal human epidermal melanocyte (NHEM 2) cells (C-12402) from PromoCell<br>Telomerase-immortalized normal human fibroblast (NHF1) cells, routinely used in the lab as described earlier, PMID: 29980679, and which were originally derived (and obtained) from Dr. William Kaufmann, University of North Carolina
Authentication	No cell lines were authenticated.
Mycoplasma contamination	The cell lines were not tested for Mycoplasma contamination
Commonly misidentified lines<br>(See ICLAC register)	No commonly misidentified lines were used in this study
